# Opening the Treasure Chest: The Current Status of Research on *Brassica oleracea* and *B. rapa* Vegetables From *ex situ* Germplasm Collections

**DOI:** 10.3389/fpls.2021.643047

**Published:** 2021-05-20

**Authors:** Katja Witzel, Anastasia B. Kurina, Anna M. Artemyeva

**Affiliations:** ^1^Leibniz Institute of Vegetable and Ornamental Crops, Großbeeren, Germany; ^2^Federal Research Center the N.I. Vavilov All-Russian Institute of Plant Genetic Resources (VIR), St. Petersburg, Russia

**Keywords:** *Brassica*, genebank, glucosinolate, phenotyping, vegetables

## Abstract

Germplasm collections reflect the genetic variability in crops and their wild relatives. Hence, those genetic resources are tremendously valuable for breeders and researchers, especially in light of climatic change and stagnant crop production rates. In order to achieve improvements in crop production and end-use quality, favorable traits and donor alleles present in germplasm collections need to be identified and utilized. This review covers recent reports on the utilization of germplasm material to isolate genotypes of *Brassica oleracea* and *B. rapa* vegetables, focusing on high nutrient use efficiency, accumulation of biologically active metabolites, pest resistance, and favorable phenotypic appearance. We discuss the current state of *Brassica* vegetable collections in genebanks and summarize studies directed to the molecular characterization of those collections.

## Introduction

National and international botanical genebanks collect, conserve, investigate, and provide genetic resources of plant species. It is crucial to preserve plant materials, such as landraces, old and advanced cultivars, inbred and double haploid lines, and hybrid populations, and to identify their characteristics. *Ex situ* conservation is most efficient in terms of ease for users to access the plant material and because of its cost efficiency. Genebanks have the task of providing precisely classified living plant material for basic research and various applied research areas, such as plant breeding (Halewood et al., 2020). By integrating morphological and anatomical analysis with molecular fingerprinting approaches, important questions about the evolution of plant diversity, species boundaries and intra-species variability are addressed and form an essential basis for reliable classification and identification of plants. More recently, high-throughput genotyping, coupled with phenotype characterization, has been introduced as a next step for efficiently mining germplasm material (Nguyen and Norton, 2020). Ideally, quality traits should also be provided for each accession, such as productivity, earliness, stress resistance, and biochemical composition (Davies and Allender, 2017), in addition to epiphenotype, the characterization of the plant-environment relationship. Hammer et al. (2018) highlight the importance of both *ex situ* and *in situ* local biodiversity conservation for a range of vegetable species, and describe the evolution of broccoli (*Brassica oleracea* var. *italica*) cultivars from a landrace. Genebanks are a valuable resource for providing plant accessions with a high level of resistance to biotic and abiotic stresses, valuable biochemical profiles or preferable sensory traits in an era of large climate changes (Pignone and Hammer, 2013; Anglin et al., 2018).

Cultivars of *Brassica* species are horticulture and field crops worldwide. They are vegetable, oil, fodder, green manure or spice plants. Brassicaceous vegetables belong to the taxa *B. oleracea* (cabbage, broccoli, cauliflower, kale, Brussels sprouts, collard greens, savoy, kohlrabi, and Chinese kale), *B. rapa* (turnip, mizuna, napa cabbage, cime di rapa, turnip rape), *B. napus* (swede), and *B. juncea* (Indian mustard), etc. These plants are grown for their root (turnip, rutabaga/swede), their swollen stem base (kohlrabi), their leaves (cabbage, kale, pak choi) or their inflorescence (cauliflower, broccoli). The importance of this class of vegetables to human health lies not only in their contribution to the vitamin and mineral components of the human diet, but also in the beneficial effects of their numerous classes of plant secondary metabolites. Just as for crops generally, maintaining their productivity in the light of increasing climate change and the dynamically evolving pest and pathogen communities represents major challenges requiring the genetic diversity in genebank accessions (Table 1).

**TABLE 1:** Coverage of *Brassica* germplasm in genebanks.

Species name	Number of accessions
*B. oleracea*	14,700
*B. napus*	8,378
*B. rapa*	7,486
*B. juncea*	4,649
*B. carinata*	732
*B. nigra*	726
*B. campestris*	455
*B. tournefortii*	185
*B. cretica*	121
*B. incana*	78
*B. fruticulosa*	72
*B. villosa*	49
*B. insularis*	47
*B. repanda*	40
*B. rupestris*	35

*The 15 most abundant *Brassica* species were retrieved from Genesys database (www.genesys-pgr.org, April 2021).*

After briefly outlining the importance of *Brassica* vegetables in human nutrition, the current state of international efforts in *Brassica* vegetable collections will be discussed. Subsequently, we will summarize different quality traits that represent possible breeding targets for crop improvement and were the focus of recent research efforts, and pinpoint strategies to overcome current shortcomings in applying genebank material for crop improvement. This review focuses on developments and achievements between 2009 and 2021 in four areas where genebank accessions have been employed: to analyze nutrient use efficiency, to gain insight into the accumulation of special metabolites, to isolate sources of pest resistance and for phenotype studies.

## Nutritional and Biologically Active Compounds in *Brassica* Vegetable Crops

In developed societies, the dietary and medicinal properties of food has become the most important factors when choosing food. *Brassica* crops have a high content of water and low fat, and consequently a low caloric content. They are characterized by a relatively high level of carbohydrates, proteins that include essential amino acids, and many mineral elements. The high value of *Brassica* crops for human nutrition is primarily determined by their wide variety of biologically active compounds – enzymes, pigments, vitamins, and secondary metabolites (Manchali et al., 2012). Breeding for biologically active compounds has been considered a priority (Prange and Hewett, 2003). Fresh *Brassica* vegetables contain antioxidants, such as vitamins C and E, carotenoids and antioxidant enzymes such as catalase, superoxide dismutase and peroxidase (Singh et al., 2010). Furthermore, they are also rich in beneficial plant metabolites, including glucosinolates, anthocyanins, flavonoids, terpenes, S-methylcysteine sulfoxide, coumarins, and other minor compounds (Manchali et al., 2012; Neugart et al., 2018).

The beneficial effects of *Brassica* vegetables on human health have been partially linked to these phytochemicals, which may act at different and complementary levels. They prevent oxidative stress, induce detoxification enzymes, stimulate the immune system, inhibit malignant transformation and carcinogenic mutations, and reduce proliferation of cancer cells (Boivin et al., 2009; Herr and Buchler, 2010; Kestwal et al., 2011). Consumption of cruciferous vegetables appears to lower the risk for some kinds of cancers, including renal, prostate and possibly colorectal cancers. Consumption of vegetables, including *Brassica* species, has been strongly associated with a reduced risk of chronic conditions such as cardiovascular disease, diabetes, Alzheimer’s disease, cataracts and age-related functional decline (Cohen et al., 2000; Knekt et al., 2002; Higdon et al., 2007; Sergentanis et al., 2018).

Greater fundamental knowledge of the accumulation of nutrients and biologically active substances by plants is required to permit the development of a strategy for creating new varieties of Brassicaceae family vegetables with improved consumer properties. Genebank germplasm represents a valuable natural genetic resource that can be exploited for the improvement of traits related to plant growth, pest resistance, and nutrient use efficiency.

## Current Status of *B. Oleracea* and *B. Rapa* Vegetable Collections

Wheat, rice, barley and maize account for a total of about 1,656,000 genebank accessions (retrieved from http://www.fao.org/wiews/en/on October 15th 2020). In Europe, around 31% of agricultural production value is represented by less than 20 crops. These include the four main European staple crops (wheat, potato, maize, and barley), several vegetable crops (tomato, sugar beet, cucumbers, melons/watermelons, *Brassicas*, and onion) and fruits (apples, citrus). Around one million accessions of these crops, held in worldwide genebanks, are listed in the Genesys online catalog, among them 31,653 accessions of *Brassica* vegetables^1^. For example, germplasm of broccoli (*B. oleracea* var. *italica*) is hold in genebank in the United Kingdom (448 accessions), Taiwan (316 accessions), Russia (172 accessions), United States (165 accessions), Germany (136 accessions), Italy (105 accessions), India (94 accessions), Australia (60 accessions), and Spain (56 accession), among others (see text footnote 1, April 2021). Most biological diversity of *Brassica* crops is now conserved *ex situ* in genebanks and breeders’ materials. The largest national collections of *B. oleracea* are located at the Russian N.I.Vavilov Institute of Plant Genetic Resources (VIR) and the German Leibniz Institute of Plant Genetics and Crop Plant Research (IPK), while the largest *B. rapa* collections are maintained at the Australian Grains Genebank (AGG) and VIR. The history of the 97-years old Russian *Brassica* collection, and results and perspectives of its study were summarized recently (Artemyeva et al., 2019). Botanical and morphological descriptions of four *Brassica* species from the German national collection were presented by Gladis and Hammer (1992).

The formation of Russian state worldwide Brassicaceae collection kept at VIR had begun in 1923 after N. I. Vavilov‘s visit of West-European countries, United States, and Canada (1921-1922). After that, collecting missions had continued in Russia and all centers of plant origin and diversity worldwide. 1,500 accessions of vegetable *Brassicas* and 450 oilseed accessions were collected by N. I. Vavilov himself and his colleagues until 1940. As a result of the complex evaluation in the different ecologo-geographical zones of Russia and the phylogenetic studies of subspecies, varieties, forms, cultivar groups using many biological traits, descriptors were developed for *B. oleracea*, *B. rapa*, *B. napus*, *Raphanus sativus*, *B. juncea*, *Sinapis alba, S. nigra*, *S. arvensis*, among others (Sinskaya, 1928; Lizgunova, 1984). On this basis, Lizgunova divided the white cabbages into 7 eco-geographical groups and 33 cultivar types, cauliflower and broccoli into 7 groups and 21 cultivar types, savoy into 5 groups and 16 types, etc. Later, Artemyeva divided the Chinese cabbages (*B. rapa* ssp. *pekinensis*) into 14 cultivar types (Artemyeva, 2004). Nowadays the VIR Brassicaceae collection consists of 10,997 accessions from 11 genera and 32 species: vegetable, fodder, oilseed, spicy, ornamental crops from 98 countries. The collection includes the accessions of different status: wild species, landraces (30%), old and advanced breeding cultivars (58%), inbred and double haploid lines, hybrid populations, and mapping populations (12%).

The genetic diversity of local and traditional *Brassica* cultivars can be used to genetically improve plants, expand their base and create new cultivars adapted to growing in less favorable environmental conditions. Currently, the international movement of genetic resources takes place for a variety of purposes and in different forms: exchange of *ex situ* germplasm accessions, sales of varietal seeds, exchange through seed companies, and management of *in situ* storage. Within the framework of the international exchange of genebank accessions, several tens of thousands of transactions are carried out annually; such exchange plays an important role in the conservation, research and development of genetic resources. Continuous availability for research and development is a prerequisite for improving crops, including *Brassica* crops. The genetic resources of *Brassica* plants have the potential to create a variety of traits that can help address potential future challenges, such as the need for crops to adapt to changing climatic conditions or disease and insects resistance. Therefore, continued access to them is necessary to meet the growing demand for food, as well as access to neglected and underutilized crops, given their important role in nutrition. Until now, several domesticated and wild Brassica species have been subjected to genome analysis for the identification of beneficial alleles (Saad et al., 2021). Merging available genome information, reverse genetics resources and genome editing tools with the diversity in germplasm pools is the most promising strategy for crop improvement.

Most conserved accessions are labeled with basic descriptors of morphological traits and information on the origin of collected material. For example, the VIR passport database is based on 28 characteristics and includes all accessions from the collections; the characterization and evaluation databases include 60–90% accessions depending of crops, while also a separate trait database is operated for characteristics of genetic and breeding interest. Hence, a proportion of material consists of unwanted duplications that need to be identified and minimized within and between collections (Solberg et al., 2018; Palme et al., 2020). A further issue is the taxonomic misclassification of accessions. Molecular characterization of plant material has become a valuable tool to increase the efficiency of genebank management and to identify duplicated or misclassified accessions (Mason et al., 2015; Pelc et al., 2015; Koh et al., 2017; Stansell et al., 2018; Yousef et al., 2018). The genetic molecular resources represented by Brassicaceae seed collections have been reviewed (Knee et al., 2011). In order to assess the genetic diversity present in *Brassica* collections, amplified fragment length polymorphisms have been studied in 50 accessions of *B. oleracea* (van Hintum et al., 2007), 17 accessions of *B. oleracea* convar. *acephala* (Christensen et al., 2011) and 20 accessions of *B. oleracea* var. *capitata* (Faltusova et al., 2011). Sequence-specific amplification polymorphism has been shown to be useful for phylogenetic analysis of the *B. rapa* core collection of VIR, encompassing 96 accessions (Budahn et al., 2013). Others have used single-sequence repeat markers to investigate genebank germplasms, such as 25 accessions of different *B. oleracea* subspecies (El-Esawi et al., 2016) and 239 accessions of *B. rapa* divided into the main morphotypes (Zhao et al., 2010), as well as several core collections from the VIR *B. oleracea* and *B. rapa* accessions: 18 Russian local white cabbage accessions by SSRs were divided into Northern and Central Russian, Dutch and Central European, Southern European cultivars, when the level of single-sequence repeat DNA polymorphism within the Russian gene pool was higher than within the European gene pool (Artemyeva et al., 2006), 96 accessions of *B. rapa* (Artemyeva et al., 2008), 50 accessions from different *B. oleracea* varieties – cabbages, cauliflower, kales (Artemyeva et al., 2009) and 40 broccoli accessions were divided into four clusters according to vegetation period length, curd size and sprout development (Fateev and Artemyeva, 2020).

In order to determine genotype-phenotype correlations in germplasm collections, association mapping using mostly SSR markers have been performed in *B. rapa, B. oleracea* and *R. sativus* accessions with respect to agronomic traits and morphological characteristics: bolting time, growth related traits, morphological characters connected with quality and productivity (Artemyeva et al., 2013) as well as biochemical traits: content of dry matter, sugars, protein, ascorbic acid, carotenoids, chlorophylls (Artemyeva et al., 2016, 2017). For instance, in *B. rapa* the most important loci are located on the top of chromosome A02, on the bottom of A03, in the higher region of A10 that correspond to *BrFLC2, BrFLC5, BrFLC1* positions, supporting the importance of flowering time for the development of many morphological and biochemical traits in *Brassica* crops.

## Research Focus of the Last Years in Utilizing Germplasm Material

The large potential of germplasm material, sometimes referred to as “diamond in the rough,” needs extensive work to make use of accessions more feasible. To establish the true value of genetic resources, broad evaluation is required by curators with respect to desired plant characteristics, e.g., agronomic, nutritional or resistance traits. For example, more than 500 vegetable *Brassica* accessions per year have been trialed at five VIR experimental stations.

Most genebanks have applied trait analyses. For example, systematic study of its worldwide collection of cultivated plants has been conducted at the VIR Department of Biochemistry since 1922, as summarized by Ivanov et al. (1938), Ermakov et al. (1972), Ermakov and Arasimovich (1961), and many others. Study of the biochemical composition of the Brassicaceae collection at VIR began in 1933. Accessions of cole crops were assessed by dry matter, sugars, protein, ascorbic acid, carotenoids, and chlorophylls. In 1951, studies on varieties of cabbage were performed to assess ascorbic acid, dry matter, sugars, fiber, mineral elements, carotene, and proteins, and the dynamics of accumulation and consumption of nutrients. Later, the genetic and geographical variabilities of the accumulation of biochemical compounds were established including vitamins, pigments, mustard oils, in cabbage (Lukovnikova, 1959; Lukovnikova and Lizgunova, 1965), turnips and rutabagas (Solovyeva et al., 2013), and radishes (Shebalina and Sazonova, 1985). In the 1970s, studies of the biochemical composition and varietal diversity of the *Brassica* collection were deepened (Lizgunova et al., 1978). In recent years, the focus of attention has been on accumulation of the major biochemical components and biologically active substances (Artemyeva et al., 2006; Solovyova et al., 2014).

### Nutrient Use Efficiency

The optimal use of essential nutrients is very important for plant production in terms of productivity and sustainability. Increasing nutrient use efficiency is critical to improve crop yield, reduce fertilizer demand and alleviate environmental pollution. In order to determine the natural variation for shoot zinc content, 376 accessions of *B. oleracea* and 74 commercial cultivars were investigated, revealing high variation and also an interaction with soil phosphorus availability (Broadley et al., 2010). The same set of lines was tested for potassium use efficiency in the field and in the greenhouse, and a twofold variation in shoot potassium content was found, encouraging further breeding approaches (White et al., 2010).

Another aspect of plant nutrition is the use of organic farming production systems. Here, cultivars with high nutrient use efficiency are in demand due to the reduced input of fertilizers. Yousef et al. (2015) monitored the growth of 178 *B. oleracea* var. *botrytis* accessions under organic and conventional farming conditions, and identified genotypes that are suitable as parental lines for breeding under organic farming conditions.

### Accumulation of Metabolites

As outlined above, *Brassica* vegetables are rich in biologically active metabolites that account for their health-promoting effects. Hence, there is a special interest in increasing the accumulation of these value-adding compounds in vegetables. Glucosinolates have been a focus of research activity for many years and numerous studies have evaluated germplasm material genotypes of diverse geographical origin.

In a field trail, a total of 146 cabbage (*B. oleracea* var. *capitata*) genotypes were analyzed and accessions identified with increased concentrations of glucosinolates (Bhandari et al., 2020). A collection of 70 Turkish white head cabbages (*B. oleracea* var. *capitata* sub. var. *alba*) grown in the field in two harvesting seasons revealed a large variation in glucosinolate composition and concentration, leading to the identification of promising genotypes for further breeding activities (Sarikamis et al., 2009).

Lee et al. (2013) profiled 48 *B. rapa* turnips for glucosinolate accumulation and found a grouping of genotypes. Similarly, the glucosinolate patterns and antioxidant activities of 62 varieties of Chinese cabbage (*B. rapa* ssp. *pekinensis*) were determined and several genotypes for further breeding were identified (Lee et al., 2014). Leaves and tubers of 16 *B. rapa* turnip accessions were assayed in parallel for their accumulation of glucosinolates and glucosinolate hydrolysis products (Klopsch et al., 2017). Besides a tissue-specific accumulation of glucosinolates and their breakdown products, the study revealed that clustering patterns of accessions based on their glucosinolate pattern differed from those based on the hydrolysis products, indicating the importance of profiling the latter bioactive compounds. The same observation was made when profiling 91 leafy *B. rapa* accessions from different subspecies (Klopsch et al., 2018). Correlations in abundance of specific glucosinolates and of their breakdown products were identified, with implications for future breeding activities.

Fifty-one accessions from 5 genera of family Brassicaceae from the VIR collection (*Sinapis alba*, *Lepidium sativum*, *Eruca sativa*, *Diplotaxis muralis*, *B. juncea*, *B. rapa*) were profiled for 21 glucosinolates. The highest content (more than 40 μM/g) was found in all accessions of white mustard (sinalbin) and in six accessions of Indian mustard (sinigrin), hence, these species should be useful for biofumigation approaches. Seventeen glucosinolates components have been determined in *B. rapa*, among them were indol glucosinolates, which are more useful for human, and three local accessions of Chinese cabbage had threefold higher levels of indole glucosinolates as compared to the remaining tested cultivars (Solovyeva et al., 2013).

A set of 19 Italian kale (*B. oleracea* var. *acephala*) was profiled for the accumulation of phenols, flavonoids, and anthocyanins, representing the basis for further nutritional use of locally adapted accessions (Lotti et al., 2018). A set of 70 white cabbage and 30 cauliflower accessions were characterized for variability of biochemical compounds at VIR; the accumulation by different genotypes of protein, sugars, ascorbic acid, carotenoids, chlorophylls, amino acids, organic acids, fatty acids, and phenolic compounds was determined. The sources of high levels of nutritive and biologically active substances were found, mostly within central Russian and Dutch white cabbage accessions, and within German and French cauliflower accessions (Solovyova et al., 2014). The connection between primary and secondary metabolism in different colored cauliflower curds was investigated and revealed closely related metabolic networks (Park et al., 2013). The complex of morphological and biochemical traits of an Italian broccoli and cauliflower landraces collection compared with F1 was studied by Branca et al. (2018) who detected a large diversity of glucosinolate, anthocyanin, carotenoids, polyphenols and ascorbic acid contents. Single sequence repeat analysis divided the collection into five clusters; all Sicilian accessions were distinct. A core collection of 168 *B. rapa* accessions of different morphotypes and origins was explored to find genetic association between markers and tocopherols, carotenoids, chlorophylls and folate (Del Carpio et al., 2011).

### Pest Resistance

Crop production is severely threatened by herbivores feeding on *Brassica* vegetables and the distribution of pests has increased in the recent years due to the expansion of rapeseed. Hence, the role of genetic sources of resistance, including landraces with high adaptability, is increasing in breeding programs. At the same time, the specificity of the human’s consumption of these crops as food requires a maximum degree of ecological safety of products, and modern ecological thinking in plant protection requires minimizing the anthropogenic impact on the components of agrobiocenosis, including useful entomofauna.

The most common pests of Brassicaceae family crops are thrips (*Thrips tabaci*), cabbage root fly (*Delia radicum*), cabbage moth (*Mamestra brassicae*), cabbage aphid (*Brevicoryne brassicae*) and diamond back moth (*Plutella xylostella*). A screen of 27 cabbage (*B. oleracea* var. *capitata*) cultivars revealed a range of preference of cabbage aphid toward the tested accessions (de Melo et al., 2013). Plant resistance tests of eight genotypes of collard greens (*B. oleraceae* var. *acephala*) against cabbage aphid revealed an interactive effect of biochemical and morphological resistance mechanisms (Canassa et al., 2020). Sources of resistance against cabbage moth were detected in a screen of 21 local and commercial cabbage varieties and interactive effects of leaf traits, head compactness, and leaf glucosinolate content were identified (Cartea et al., 2010). A set of 56 turnip (*B. rapa* ssp. *rapa*) accessions were screened for resistance against cabbage root fly under controlled conditions and in the field, leading to the isolation of resistant cultivars and resistance plant traits (Santolamazza-Carbone et al., 2017). Resistance against cabbage whitefly (*Aleyrodes proletella*) was tested in a collection of 432 accessions of *B. oleracea* and its wild relatives. It was shown that the wild relatives exhibited an earlier resistance than the breeding cultivars, which is probably due to the earlier formation of trichomes (Pelgrom et al., 2015). The effect of epicuticular waxes on plant resistance against the green peach aphid (*Myzus persicae*) was minor in twelve kale genotypes (Costa et al., 2014).

### Phenotypic Appearance

Besides health and welfare benefits, an important marketing goal of *Brassica* producers is the attractive appearance, size and shape of crops. Hence, genebank accessions are screened for phenotypic traits. Thirty kale genotypes were investigated for 44 morphological traits, revealing a limited extent of phenotypical variation albeit from a wide extent of genetical divergence (Azevedo et al., 2014). Thorwarth et al. (2018) selected 174 cauliflower accessions to gain insight into six curd-related traits. Further, they identified associations between these traits and genetic markers by genome-wide association mapping.

At VIR, a complete phenotyping of all accessions in the different eco-geographical zones of Russia is performed according to the same methodic during three years. The VIR evaluation databases include 47–55 morphological and phenological characters for vegetable crops, 18–23 characters for oilseeds, as well as separate immunological, physiological, and biochemical databases. Since pathogenic organisms spoil the phenotypic appearance of crops, trait collections have been established based on resistance/susceptibility to black rot, clubroot, downy mildew, and leaf spot disease. The range of responses was dependent on the crop, the pathogen race and geographical origin of the accessions. For instance, maximum frequency of *B. rapa* resistance to black rot originates from accessions from Central-Asian subpopulation, which is considered as the most genetically variable part of the species genepool, and to a subpopulation from Japan (Ignatov et al., 2011).

The variability of ten accessions of Chinese broccoli was investigated for morphological, biochemical, genetic properties and a large diversity was noted in this set, generally on plant habit, stem diameter and chlorophyll content (Fotev et al., 2018). Morphological and biochemical traits linked to consumer value of 96 *B. rapa* accessions from different morphotypes from the VIR core collection were screened in the field and under greenhouse conditions in the North-West Russia and in the field in South China. The study revealed correlations between characteristics, and sources for breeding and genetic markers for traits investigated (Artemyeva et al., 2017). A study of the morphological traits of the plant, leaf lamina and root of 16 local Scandinavian turnip accessions from the VIR collection allowed determination of typical (cvs. Petrovskaya, Karelskaya, Tankard yellow) and rare types (cv. Kostenevskaya), and mixed populations, as well as valuable stock for breeding projects (Kornyukhin and Artemyeva, 2017).

## Conclusion

Genebanks have many tasks and challenges (Fu, 2017; Diez et al., 2018). At present, the high-throughput use of omic tools for extensive germplasm collections is challenging due to the laborious sample preparation (nucleic acids, proteins, and metabolites), as well as suboptimal handling and analysis approaches of large-scale datasets. Further, unharmonized laboratory-specific analytical techniques hinder the exchange of results between labs. However, assimilating large amounts of genome, expression and metabolite data, in conjunction with phenotype information, will truly accelerate breeding processes and increase our understanding of the molecular background of trait formation. These enormous efforts need to be supported by increased public funding. Although the public awareness on safeguarding the agricultural and horticultural heritage has increased in recent years, funding of germplasm banks did not catch up with the demands, currently covering basically permanent activities (collect, inventory, and propagate, etc.), but insufficiently financing genome and phenome investigations. The utilization of *Brassica* vegetable germplasm collections to isolate promising genotypes for breeding activities has been achieved successfully. However, there is still underutilization of germplasms, and the link between conservation and use of collections must be strengthened further. Recent activities aimed at developing user-friendly databases to aid the access to germplasm collections, ultimately centralizing database management. Currently, besides international platforms, such as Genesys, Crop Wild Relatives Global Portal, EURISCO, New World Fruits Database, and ECPGR, also numerous national databases, such as GRIN, GBIS/I, and SeedStor are available. Centralized and detailed information about genotype, phenotype, stress resistance and metabolite accumulation of accessions would increase use of collections. Applying high-throughput molecular and phenotypic screening methods will allow the deep characterization of collections and yield valuable trait information for genebank curators as well as for researchers and breeders.

## Author Contributions

KW, AK, and AA wrote the manuscript. All authors contributed to the article and approved the submitted version.

## Conflict of Interest

The authors declare that the research was conducted in the absence of any commercial or financial relationships that could be construed as a potential conflict of interest.

## Footnotes

1 www.genesys-pgr.org
